# The Influence of Solar Spectrum and Concentration Factor on the Material Choice and the Efficiency of Multijunction Solar Cells

**DOI:** 10.1038/s41598-019-56457-0

**Published:** 2019-12-27

**Authors:** Daniel N. Micha, Ricardo T. Silvares Junior

**Affiliations:** Centro Federal de Educação Tecnológica Celso Suckow da Fonseca, CEFET/RJ, Rio de Janeiro, 20271-110 Brazil

**Keywords:** Applied physics, Solar cells, Solar cells

## Abstract

In this work, we revisit the theoretical study on the conversion efficiency of series-connected multijunction solar cells. The theoretical method, based on the detailed balance model, is then applied to devices with 2 to 6 junctions under different illumination conditions. As results, (i) we show that the peaks in the efficiency distribution occur for recurrent values of bottom junction bandgap energy corresponding to atmospheric absorption in the solar spectrum, and (ii) we demonstrate that variations in the number of junctions, in the incident solar spectrum, and in the concentration factor lead to changes in the optimum bandgap energy set but that the bottom junction bandgap energy only changes among the recurrent values presented before. Additionally, we highlight that high conversion efficiencies take place for a broad distribution of bandgap energy combination, which make the choice of materials for the device more flexible. Therefore, based on the overall results, we propose more than a hundred III-V, II-VI and IV semiconductor material candidates to compose the bottom junction of highly efficient devices.

## Introduction

Multijunction solar cells (MJSC) are the most successful photovoltaic technology in using the solar resource efficiently. The current highest efficiency ever achieved by November 2019 is 47.1% demonstrated with a monolithic series-connected six junction solar cell under concentration^1^. However, MJSC are devices expensive to produce^2^ and for this reason they were successful in niche applications in which they are cost-effective, such as for space^3^ and in terrestrial use under concentrated sunlight^4^. On the other hand, modern techniques can drastically reduce production costs by the use of inexpensive materials^5–8^, high quality heteroepitaxy between groups II-VI, III-V, and IV materials^9–12^, the reuse of the growth substrate^13^ and the saving in epitaxy costs^7,14^, which can push MJSC to a cost level similar to flat-plate Si technology in the future^2,15^. Nonetheless, it is important to highlight that this optimistic estimative is strictly based in the high efficiencies still to be achieved for this kind of device. In this context, basic studies establishing the efficiency limits and defining the materials to be used in highly efficient devices are mandatory.

In 1961, Shockley and Queisser developed an elegant theory based on the detailed balance model to calculate the maximum efficiency of photovoltaic conversion by single *pn* junctions^16^. They found the limit to be 30% for a bandgap energy of 1.34 eV under a blackbody spectrum simulating the sun and 44% for a bandgap energy of 1.12 eV under maximum solar concentration. Their paper has been the trigger for several other theoretical studies which analyzed the conversion efficiency limit under different solar device architectures. In 1980, two independent studies conducted by Henry^17^ and De Vos^18^ found limiting conversion efficiencies for MJSC under concentrated sunlight to be 72% (1,000 suns and 36 junctions) for an optically and electrically series connected device and 86.8% (45,900 suns and infinite junctions) for an optically series-connected device with no electrical restriction, respectively. Several other investigations have followed taking into consideration more fundamental arguments such as thermodynamical backgrounds on entropy-energy generation^19–24^, including other physical effects, such as angle and energy restriction^21^, boundary conditions^25^, analyzing the influence of the series resistance^26^ and of materials dominated by other type of recombination processes, such as silicon^27^, and presenting new concepts, such as hot carrier solar cells^28^, intermediate band solar cells^29^, among others, enriching the literature and our comprehension of the photovoltaic conversion processes.

More recently, new studies on the theoretical efficiency of MJSC have taken into account the role of the optical interconnection among the junctions under the photon recycling and the luminescent coupling effects^30–37^. It is well understood that the recycling of the emitted photons by the same emitting junction increases the device open circuit voltage (V_OC_) and that the luminescent coupling among the junctions can increase the device short circuit current (I_SC_) allowing the device to approach the current matching condition. To these effects to positively contribute to the total generated power, the materials have to be limited by radiative recombination and the connection between the junctions needs to be optically transparent. Considering a more realistic scenario, Zhu *et al*.^38^ took into consideration the influence of non-radiative recombination using the internal radiative efficiency as a parameter in the efficiency calculations. They showed that conversion efficiencies are reduced and that the optimum bandgap energy sets change under the increase of the amount of non-radiative recombination.

The production of devices trying to fulfil the optical and mechanical requirements for a high-quality device, as predicted by theoretical studies, has followed up. The first demonstrated triple junction solar cell (3JSC) was a monolithic lattice matched GaInP/GaAs/Ge (1.90/1.42/0.67 eV) by Spectrolab in 2000 with an efficiency of 32.3% at concentrated sunlight^39^ (hereon the efficiencies are presented under concentrated sunlight except when explicitly mentioned). At that point, several different configurations of dual junction solar cells (2JSC) already existed fabricated with the most mature-to-produce monolithic lattice matched materials, such as GaInP/GaAs, InP/GaInAs, GaAs/GaInAsP, showing efficiencies up to 32.6%^39^. The following next decades have been marked with important innovations in the MJSC development yielding a rapid increase in their efficiencies. In 2001, Bett *et al*. demonstrated a 33.5% efficient mechanically stacked GaInP/GaInAs/GaSb (1.74/1.17/0.73 eV) 3JSC^40^. In 2005, Wanlass *et al*.^41^ presented a revolutionary new technique to produce non-lattice matched monolithic structures based on (inverted) metamorphic growth. Therefore, they showed a 37.9% effici ent monolithic metamorphic GaInP/GaAs/GaInAs (1.87/1.42/1.02 eV) 3JSC. After this new technique, the progress have followed quickly among initiatives using approaches with lattice matched materials, such as GaInP/GaInAs/Ge (1.87/1.4/0.67 eV)^42^, GaInP/GaAs/GaInNAs (1.86/1.42/1.0 eV)^43^, inverted metamorphic materials, such as GaInP/GaInAs/GaInAs (1.83/1.34/0.89 eV)^44^, and metamorphic materials, such as GaInP/GaInAs/Ge (1.74/1.17/0.67 eV)^45^. The current 3JSC highest efficiency since 2013 is 44.4% for an inverted metamorphic GaInP/GaAs/GaInAs structure developed by Sharp Co.^1^. In 2014, researchers from Fraunhofer ISE demonstrated a GaInP/GaAs//GaInAsP/GaInAs (1.88/1.42/1.12/0.74 eV) four junction solar cell (4JSC) with an efficiency of 44.7%^1,46^ based on a new technique: wafer bonding. This technique allows bandgap engineering without the need of the lattice matching constraint due to the direct bonding between crystals (the//symbol indicates where the bond is located). An improved version of this device has been further demonstrated with an efficiency of 46%^1,47^. Still in 2014, NREL also demonstrated a very high efficiency of 45.7% with a GaInP/GaAs/GaInAs/GaInAs 4JSC based on the inverted metamorphic approach^1,48^. The development of solar cells with more than four junctions is still in its very beginning. Nevertheless, the highest efficiency ever achieved up to November 2019 has been obtained with a AlGaInP/AlGaAs/GaAs/GaInAs/GaInAs/GaInAs (2.15/1.72/1.41/1.17/0.96/0.70 eV) six junction solar cell (6JSC) developed by NREL^1,49^. In addition, two results worth to mention are a 38.8% efficient wafer bonded 5JSC at 1-sun by Spectrolab^1,50^ and a 39.2% efficient 6JSC at 1-sun by NREL^1,51^.

The development history of MJSC have established an important guideline for the optimization of MJSC structures: theoretical calculations shall guide the choice of materials. However, it is possible to see an ambiguity in the definition of the optimum bandgap energy for the bottom junction (which in last instance determines the others). While some authors defend a bandgap energy of ~0.7 eV^20^, others say that ~1.0 eV would lead to the highest efficiency^41,52^ or even point both values when evaluating for different spectra^4,53^. In this context, one could blame the theoretical approach to generate different values for the optimal bandgap combination leading to the highest efficiencies, but this is not the case. Actually, both values for the bandgap energy of the bottom junction lead either to a global or a local maximum in the efficiency of MJSC. This is due to the particular illumination condition impinging in the solar cell regarding the solar spectrum and the concentration factor.

As already observed by many authors^20,38,52–54^, the molecular absorption of portions of the solar spectrum by the atmospheric gases is responsible for the reduction of the photon flux in some wavelength ranges. McMahon *et al*.^54^ correlated the disruption of the current matching in MJSC to this effect. They indicated the wavelength bands in which the efficiency is altered leading to the global and local maxima. The high energy edge of each band is of great importance, as they are the energy of the efficiency peaks. As such energies are of interest in this work, we labeled them as *E*_*A*_ = 0.52 eV, *E*_*B*_ = 0.70 eV, *E*_*C*_ = 0.93 eV, *E*_*D*_ = 1.12 eV, and *E*_*E*_ = 1.34 eV and use them hereon.

The bottom junction bandgap energies leading to the historically highest experimental efficiencies for MJSC have varied between the corresponding values of *E*_*B*_ and *E*_*C*_, as discussed. Despite McMahon *et al*.^54^ and other authors have correctly observed and explained the influence of the solar spectrum shape in the efficiency of MJSC, they did not consider that the variation in the concentration factor also has an impact in the MJSC efficiency and its optimum configuration. As it is going to be demonstrated, even for the same application (regarding spectrum and number of junctions), the optimum bandgap energy set changes with concentration. This is the reason for the divergence in the results of the different theoretical studies.

In this work, we revisit the theoretical study on the conversion efficiency of MJSC. The numerical methods used in this work (described in Methods) are based on the detailed balance model applied to MJSC with 2 to 6 junctions under several illumination conditions. Rather than focusing only in the maximum achievable efficiency, we thoroughly study the efficiency distribution in the *N*-dimensional bandgap energy space, in which *N* is the number of junctions, to investigate the more promising choice of materials in the design of highly efficient devices. As results, we reinforce previous studies conclusions showing that the highest efficiencies for terrestrial application take place for recurrent values of the bottom junction bandgap energy for any configuration regarding number of junctions and illumination condition. Moreover, we demonstrate that the optimum values for the bandgap energy sets change with the concentration factor even for the same spectrum and number of junctions, which directly impacts the device design for the same application. Finally, the observed recurrence for the best bottom junction bandgap energy allowed us to suggest more than a hundred promising materials to compose this junction in highly efficient MJSC for any terrestrial application.

## Results and Discussion

### MJSC efficiency under AM0 and AM1.5 g spectra

**Figure 1:**
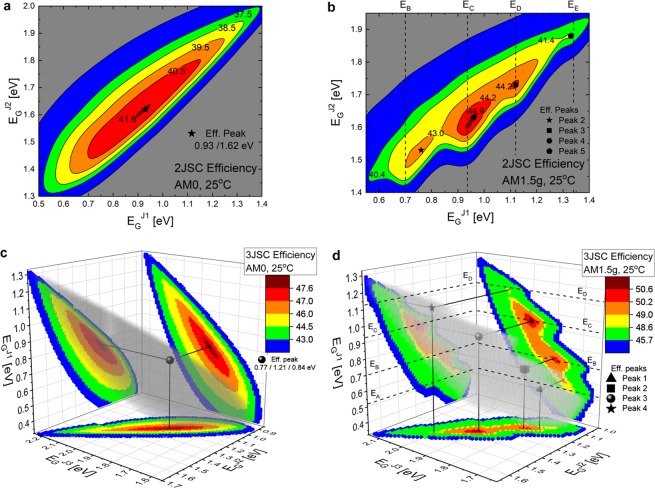
Multijunction solar cell efficiency color maps. (**a**,**b**) 2JSC efficiency (color mappings and level curves) as a function of *E*_*G*_^*J1*^ and *E*_*G*_^*J2*^ under AM0 and AM1.5 g, respectively. (**c**,**d**) 3JSC efficiency (color mappings) as a function of *E*_*G*_^*J1*^, *E*_*G*_^*J2*^ and *E*_*G*_^*J3*^ under AM0 and AM1.5 g, respectively. The efficiency peaks are marked with symbols and are numbered increasingly according to the energies *E*_*A*_ – *E*_*E*_.

Figure 1 presents color mappings and level curves for the efficiency of 2JSC and 3JSC as a function of the bandgap energies of their composing junctions for two different illumination conditions: (a) 2JSC under AM0, (b) 2JSC under AM1.5 g, (c) 3JSC under AM0, and (d) 3JSC under AM1.5 g. The junctions are numbered increasingly (*i* = 1..*N*) from the bottom to the top, i.e., *E*_*G*_^*J1*^ is the bottom junction bandgap energy. In Fig. 1c,d, the transparent gray spheres indicate the swept xyz relevant points (as described next) whereas the colorful projections in the axis planes facilitate the observation of the corresponding bandgap energies leading to such results.

From Fig. 1, it is possible to see that the efficiency for the AM0 spectrum (Fig. 1a,c) has a single peak whereas for the AM1.5 g spectrum (Fig. 1b,d), it presents a multi-peak behavior, as expected due to atmospheric absorption^53,54^. In the figures, the efficiency peaks are marked with symbols within the xyz point distribution. Furthermore, Peaks 1 to 5 in AM1.5 g show up at *E*_*G*_^*J1*^ close to the same energies *E*_*A*_ to *E*_*E*_ (the peak numbering is in accordance to the energies *E*_*A*_ – *E*_*E*_), as specified by the vertical dashed lines in Fig. 1b and the horizontal dashed lines in Fig. 1d. It is important to notice that the correlation takes place exclusively for *E*_*G*_^*J1*^.

Another result noteworthy is the broad range of bandgap energy combinations leading to high efficiencies (relevant points with efficiency higher than 90% of the efficiency peak). In Fig. 1, such regions are delineated by the contour lines separating the green and the blue areas (second last contour line in the figures), specifically at 37.5% and 40.4% for 2JSC under AM0 and AM1.5 g, respectively, and at 43.0% and 45.7% for 3JSC under AM0 and AM1.5 g, respectively. This is an important result concerning the design of highly efficient 2JSC and 3JSC, which allows an extensive choice of materials to compose the device junctions not necessarily attaining only to the bandgap energy values leading to the global efficiency peak.

**Figure 2:**
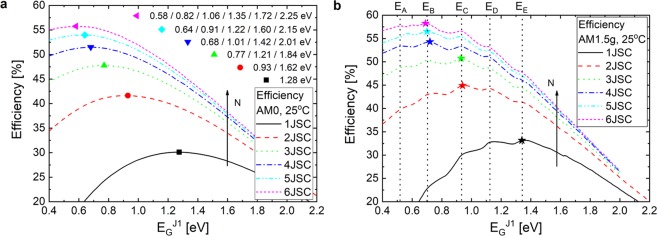
Best MJSC efficiencies for AM0 and AM1.5 g. (**a**,**b**) Dependence of the best efficiencies on *E*_*G*_^*J1*^ in MJSC with 1 to 6 junctions for AM0 and AM1.5 g spectra, respectively. In (**a**), the single peaks are marked with featured symbols and the corresponding bandgap energies are shown in the legend. In (**b**), the highest efficiencies are marked with stars and the energies *E*_*A*_ – *E*_*E*_ are marked with vertical black dashed lines.

The analysis for MJSC with more than three junctions followed. Although, as the *N*-dimensional domain with *N* > 3 is not possible to show in a single graph as done for 2JSC and 3JSC, we present an analysis of the efficiency over *E*_*G*_^*J1*^ to further evaluate the correlation between the peaks and the absorption energy bands in the solar spectrum. Figure 2 shows the dependence of the best MJSC efficiencies on *E*_*G*_^*J1*^ for (a) AM0 and (b) AM1.5 g and the number of junctions *N* varying from 1 to 6.

Over again, the same correlation between the *E*_*G*_^*J1*^ values leading to the efficiency peaks for the terrestrial spectrum and the energies *E*_*A*_ – *E*_*E*_ is observed. Moreover, from Fig. 2, it is possible to see that the highest efficiency peak is redshifted and increases as the number of junctions increases for both illumination conditions. This is expected as a result of a more balanced division of the solar photons among the junctions and to a reduction of the losses related to transmission and thermalization. Furthermore, the highest efficiency peak for the AM1.5 g spectrum is not deviated continuously as for the AM0 spectrum. Instead, the peaks are interchanged only among the *E*_*A*_ *– E*_*E*_ values.

The gain in efficiency by adding a new junction is much higher when the number of junctions is low. From 2JSC to 3JSC under AM1.5 g, the maximum efficiency raises by 5.8% in absolute (13% relative) whereas from 5JSC to 6JSC only 1.7% (3% relative) is added. By a first estimative (whose methods to find are described in Methods), the maximum efficiency of a 10JSC is around 61%, a gain of only 2.8% in absolute (4.8% relative) compared to a 6JSC, for example. Thus, the stacking of too many junctions in a device to increase the efficiency even further shall be evaluated from the cost-effectiveness point-of-view.

### Efficiency of MJSC under concentrated AM1.5d spectrum

**Figure 3:**
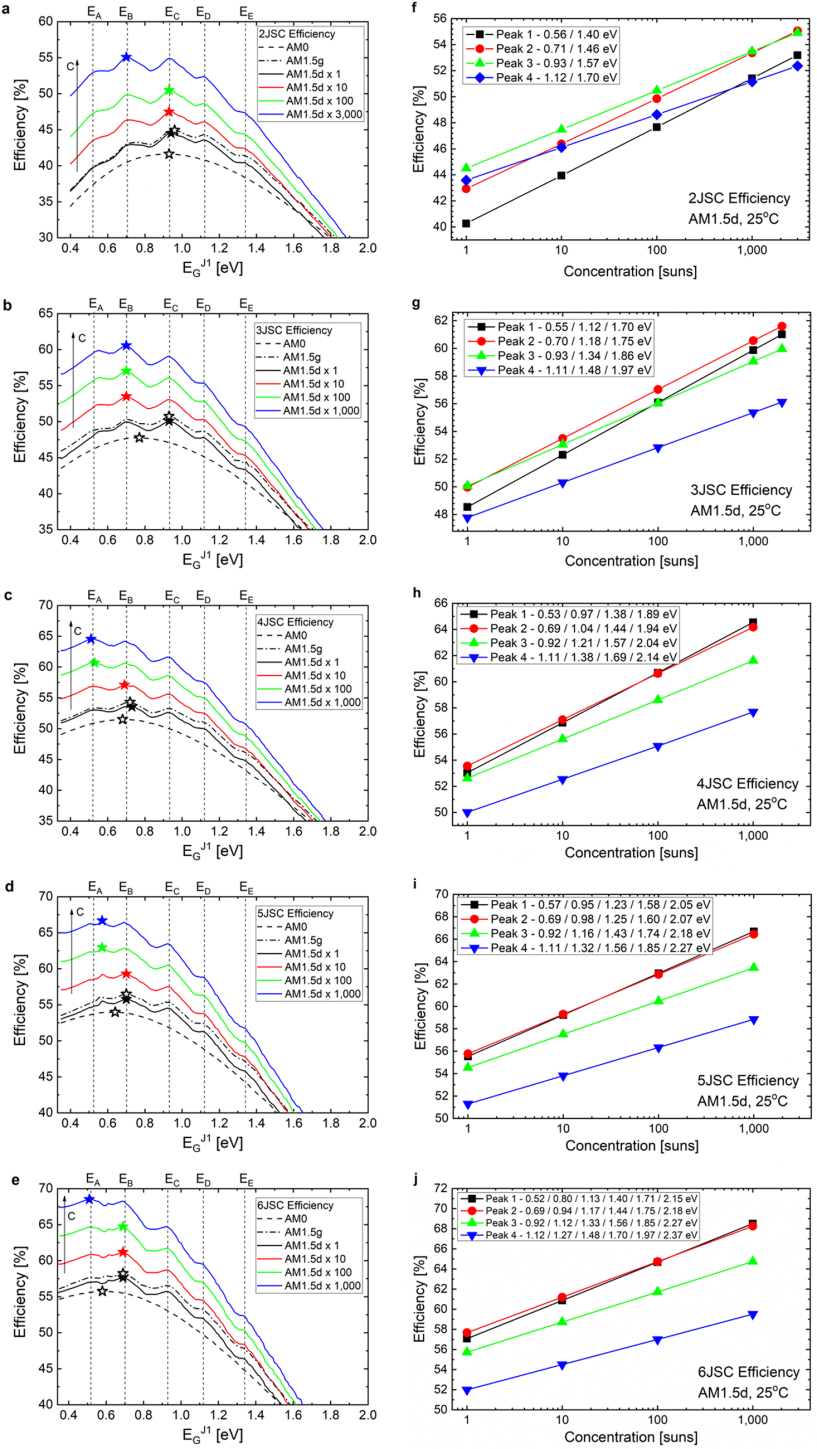
Efficiency of MJSC under concentrated AM1.5d spectrum. (**a**–**e**) Dependence of the MJSC best efficiency on *E*_*G*_^*J1*^ for different illumination conditions for 2JSC, 3JSC, 4JSC, 5JSC, and 6JSC, respectively. (**f**–**j**) Dependence of the MJSC peak efficiency on the concentration factor for AM1.5d spectrum for 2JSC, 3JSC, 4JSC, 5JSC, and 6JSC, respectively. The points in the figures on the right-hand side are related to the peaks present in the figures on the left-hand side.

The occurrence of multi-peaks in the MJSC efficiency distribution and the recurrence of the peak positions for values of *E*_*G*_^*J1*^ close to the energies *E*_*A*_ – *E*_*E*_also take place for the AM1.5d spectrum under concentration, as can be seen by the colorful solid lines in Fig. 3 for (a) 2JSC, (b) 3JSC, (c) 4JSC, (d) 5JSC, and (e) 6JSC. Moreover, it is possible to see that the highest efficiency peak for AM1.5d, marked with colorful solid stars in the figures, changes depending on the concentration factor *C*, but only among the most recurrent values for the best *E*_*G*_^*J1*^. To further investigate this behavior, Fig. 3 also shows how the efficiency peak values depend upon the concentration factor for (f) 2JSC, (g) 3JSC, (h) 4JSC, (i) 5JSC, and (j) 6JSC.

From Fig. 3, it is possible to notice that the highest efficiency is also redshifted and increases logarithmically under concentration. To understand such relations, we shall explore the dependence of the conversion efficiency *η* on the concentration factor *C*, as described by Eq. (1)^55^:

1 $$\eta (C)=\eta (1)+\frac{(kT/q)}{{V}_{OC}^{MJSC}(1)}ln(C)$$

in which ${V}_{OC}^{MJSC}(1)$ is the MJSC open-circuit voltage for 1-sun, *k* is the Boltzmann constant, *q* is the electronic charge and *T* is the cell temperature. As Eq. (1) indicates, the higher slopes are expected to take place for the bandgap energy combinations with lower ${V}_{OC}^{MJSC}(1)$. In fact, from Fig. 3, it is possible to see that the steeper increases are for the combinations in which the bandgap energies are lower which, in fact, lead to lower ${V}_{OC}^{MJSC}$. This tendency shifts the efficiency peak to low *E*_*G*_^*J1*^ (and all other *E*_*G*_^*Ji*^) values for high concentration.

Peak 1 (depicted as black squares in Fig. 3f–j) is the highest one for 4JSC under concentrations above 60 suns, for 5JSC under concentrations above 20 suns, and for 6JSC under concentrations above 160 suns. For the same devices, Peak 2 (red circles) is the highest one at the complementary concentration ranges, for 2JSC under concentrations above 1600 suns and under any concentration factor for 3JSC. Peak 3 (green triangles) is relevant under two conditions: it is the highest peak for 2JSC under concentrations below 1600 suns and it is the second highest peak for 3JSC under concentrations below 100 suns. Peak 4 (blue diamonds) and Peak 5 (not in the figures) do not appear as the highest peak in any MJSC configuration. However, Peak 4 still shows up in the graphs of Fig. 3 because it fills the requirement of high efficiency (>90% of the highest efficiency peak). Nonetheless, it plays an important role for low concentration in the 2JSC configuration.

### Summary of MJSC configurations leading to the highest efficiencies

Table 1 summarizes the highest efficiencies of the MJSC studied in this work under several illumination conditions regarding spectrum and concentration factor. For each condition, the bandgap energy combinations of the peaks leading to efficiency higher than 90% are shown.

**Table 1:** Summary of the MJSC highest efficiencies and its configurations.

Number of Junctions – *N*	Spectrum	*η*_*Peak*_ (%)	Bandgap Energies *E*_*G*_^*J1*^/../*E*_*G*_^*JN*^ (eV)
2	AM0	41.63	0.93/1.62
	AM1.5 g	44.93	0.96/1.63
		44.36	1.12/1.73
		43.25	0.76/1.53
		41.51	1.33/1.88
	AM1.5d × 1	44.51	0.93/1.57
		43.58	1.12/1.70
		42.94	0.71/1.46
		40.26	0.56/1.40
	AM1.5d × 100	50.48	0.93/1.57
		49.85	0.71/1.46
		48.62	1.12/1.70
		47.67	0.56/1.40
	AM1.5d × 3,000	55.06	0.71/1.46
		54.90	0.93/1.57
		53.19	0.56/1.40
		52.36	1.12/1.70
3	AM0	47.77	0.77/1.21/1.84
	AM1.5 g	50.74	0.93/1.36/1.90
		50.31	0.71/1.21/1.81
		49.03	0.58/1.17/1.77
		48.69	1.11/1.49/2.00
	AM1.5d × 1	50.07	0.93/1.34/1.86
		49.97	0.70/1.18/1.75
		48.73	0.58/1.15/1.73
		47.78	1.11/1.48/1.97
	AM1.5d × 100	57.03	0.70/1.18/1.75
		56.10	0.55/1.13/1.71
		56.05	0.93/1.34/1.86
		52.84	1.11/1.48/1.97
	AM1.5d × 1,000	60.56	0.70/1.18/1.75
		59.87	0.55/1.12/1.70
		59.06	0.93/1.34/1.86
		55.37	1.11/1.48/1.99
4	AM0	51.50	0.68/1.01/1.42/2.01
	AM1.5 g	54.30	0.72/1.12/1.50/2.01
		53.36	0.54/1.00/1.43/1.95
		53.32	0.93/1.23/1.60/2.09
		51.16	1.11/1.40/1.72/2.19
	AM1.5d × 1	53.55	0.69/1.04/1.44/1.94
		53.04	0.53/0.97/1.38/1.89
		52.61	0.92/1.21/1.57/2.04
		50.02	1.11/1.38/1.69/2.14
	AM1.5d × 100	60.69	0.53/0.97/1.38/1.89
		60.63	0.69/1.04/1.44/1.94
		58.61	0.92/1.21/1.57/2.04
		55.08	1.11/1.38/1.69/2.14
	AM1.5d × 1,000	64.54	0.51/0.96/1.37/1.88
		64.17	0.69/1.04/1.44/1.94
		61.62	0.92/1.21/1.56/2.03
5	AM0	53.97	0.64/0.91/1.22/1.60/2.15
	AM1.5 g	56.50	0.70/1.00/1.31/1.66/2.14
		56.09	0.55/0.95/1.24/1.61/2.10
		55.46	0.92/1.17/1.45/1.77/2.23
		52.47	1.11/1.34/1.58/1.88/2.32
	AM1.5d × 1	55.77	0.70/1.00/1.28/1.63/2.09
		55.53	0.57/0.95/1.23/1.58/2.05
		54.54	0.92/1.16/1.43/1.74/2.18
		51.29	1.11/1.32/1.56/1.85/2.27
	AM1.5d × 100	62.96	0.57/0.94/1.22/1.57/2.04
		62.86	0.69/0.98/1.25/1.60/2.07
		60.48	0.92/1.16/1.43/1.74/2.18
	AM1.5d × 1,000	66.69	0.57/0.94/1.22/1.57/2.04
		66.44	0.69/0.98/1.25/1.60/2.07
		63.45	0.92/1.16/1.43/1.74/2.18
6	AM0	55.76	0.58/0.82/1.06/1.35/1.72/ 2.25
	AM1.5 g	58.23	0.69/0.96/1.2/1.48/1.8/2.25
		57.67	0.52/0.8/1.13/1.41/1.74/2.2
		56.58	0.93/1.14/1.37/1.6/1.91/2.34
		53.23	1.12/1.28/1.5/1.73/2.01/2.43
	AM1.5d × 1	57.66	0.69/0.94/1.17/1.44/1.75/2.18
		57.07	0.54/0.8/1.13/1.4/1.71/2.15
		55.73	0.92/1.13/1.34/1.57/1.86/2.28
		51.99	1.12/1.27/1.48/1.7/1.97/2.37
	AM1.5d × 100	64.73	0.69/0.94/1.17/1.44/1.75/2.18
		64.68	0.52/0.79/1.1/1.38/1.69/2.14
		61.72	0.92/1.12/1.33/1.56/1.85/2.27
	AM1.5d × 1,000	68.51	0.51/0.78/1.07/1.37/1.68/2.13
		68.26	0.69/0.94/1.17/1.44/1.75/2.18
		64.74	0.92/1.12/1.33/1.56/1.85/2.27

Bandgap energy combinations leading to the peak efficiencies for MJSC with 2 to 6 junctions under different illumination conditions. Only the peaks with efficiency higher than 90% of the maximum efficiency for each condition are shown.

### Choice of materials for the bottom Junction of MJSC

The invariance of the *E*_*G*_^*J1*^ values leading to the multiple efficiency peaks for terrestrial solar spectra in several MJSC configurations motivates the indication of materials to compose the bottom junction. In Table 2, we propose a list of promising materials for this purpose. The material parameters are in accordance with Vurgaftman *et al*.^56^ for III-V semiconductors, with Kudrawiec^57^ for nitrides semiconductor, and with Wei and Zunger^58^ for II-VI semiconductors. The III-V materials are presented in reference to a substrate just for orientation of the lattice parameter. Along with that, we remind the innovative technologies developed in the last two decades to allow mechanical integration of different materials either with or without the necessity of lattice matching, such as the (inverted) metamorphic growth and the direct bonding. For such reason, the comments column indicates whether the proposed material is lattice matched (LM) or lattice mismatched (LMM) to the reference substrate with a mismatching up to 3.0%. For the II-VI semiconductors, two cases are considered. In the first, no reference substrate is designated as the materials can be grown in any type of substrate (glass or plastic) in its polycrystalline form. However, in the second case, an III-V or IV semiconductor substrate is considered. This is based on some important demonstrations of integrating monocrystalline monolithic II-VI to such materials for solar cells that deserve attention^11,12,59,60^. Other possibilities of LMM materials within these systems are also possible and are not shown here but can be obtained by interpolation of the data shown elsewhere^56–58^ as well as other promising materials, such as perovskites, as Hörantner *et al*.^8^ point out.

**Table 2:** Materials for the bottom junction of MJSC for use in terrestrial applications.

Reference substrate	*E*_*G*_^*J1*^	Material for bottom junction	Comments
III-V on III-V or IV substrate			
GaAs	*E*_*C*_ = 0.93 eV	In_0.11_Ga_0.89_N_0.04_As_0.96_	LM
		GaAs_0.71_Sb_0.29_	LMM (2.3%)
		In_0.36_Ga_0.64_As	LMM (2.6%)
	*E*_*B*_ = 1.12 eV	In_0.05_Ga_0.95_N_0.018_As_0.982_	LM
		GaAs_0.84_Sb_0.16_	LMM (1.3%)
		In_0.21_Ga_0.79_As	LMM (1.5%)
	*E*_*A*_ = 1.34 eV	In_0.01_Ga_0.99_N_0.003_As_0.997_	LM
		GaAs_0.96_Sb_0.04_	LMM (0.3%)
		In_0.05_Ga_0.95_As	LMM (0.4%)
GaSb	*E*_*E*_ = 0.52 eV	In_0.16_Ga_0.84_As_0.14_Sb_0.86_	LM
		InSb_0.34_P_0.66_	LMM (0.3%)
		In_0.92_Al_0.18_As	LMM (1.1%)
		InAs_0.83_P_0.17_	LMM (1.1%)
		In_0.76_Ga_0.24_As	LMM (2.2%)
		In_0.37_Ga_0.63_Sb	LMM (2.3%)
	*E*_*D*_ = 0.70 eV	In_0.02_Ga_0.98_As_0.02_Sb_0.98_	LM
		In_0.05_Ga_0.95_Sb	LMM (0.3%)
		InSb_0.25_P_0.75_	LMM (1.2%)
		In_0.83_Al_0.17_As	LMM (1.7%)
		InAs_0.65_P_0.35_	LMM (1.7%)
	*E*_*C*_ = 0.93 eV	Al_0.17_Ga_0.83_As_0.01_Sb_0.99_	LM
		Al_0.14_Ga_0.86_Sb	LMM (0.1%)
		InSb_0.15_P_0.85_	LMM (2.2%)
		In_0.73_Al_0.27_As	LMM (2.4%)
		InAs_0.42_P_0.58_	LMM (2.4%)
	*E*_*B*_ = 1.12 eV	Al_0.32_Ga_0.68_As_0.03_Sb_0.97_	LM
		Al_0.29_Ga_0.71_Sb	LMM (0.2%)
	*E*_*E*_ = 1.34 eV	Al_0.46_Ga_0.54_As_0.04_Sb_0.96_	LM
		Al_0.53_Ga_0.47_Sb	LMM (0.3%)
Ge	*E*_*C*_ = 0.93 eV	In_0.12_Ga_0.88_N_0.04_As_0.96_	LM
		GaAs_0.71_Sb_0.29_	LMM (2.2%)
		In_0.36_Ga_0.64_As	LMM (2.5%)
	*E*_*B*_ = 1.12 eV	In_0.058_Ga_0.942_N_0.017_As_0.983_	LM
		GaAs_0.84_Sb_0.16_	LMM (1.2%)
		In_0.21_Ga_0.79_As	LMM (1.4%)
	*E*_*E*_ = 1.34 eV	In_0.02_Ga_0.98_N_0.002_As_0.998_	LM
		GaAs_0.96_Sb_0.04_	LMM (0.2%)
		In_0.05_Ga_0.95_As	LMM (0.3%)
InAs	*E*_*A*_ = 0.52 eV	In_0.26_Ga_0.74_As_0.32_Sb_0.68_	LM
		InAs_0.77_Sb_0.07_P_0.16_	LM
		InSb_0.34_P_0.66_	LMM (0.3%)
		In_0.92_Al_0.18_As	LMM (0.5%)
		InAs_0.83_P_0.17_	LMM (0.5%)
		In_0.76_Ga_0.24_As	LMM (1.6%)
	*E*_*D*_ = 0.70 eV	In_0.09_Ga_0.91_As_0.17_Sb_0.83_	LM
		InAs_0.61_Sb_0.12_P_0.27_	LM
		In_0.05_Ga_0.95_Sb	LMM (0.9%)
		InSb_0.25_P_0.75_	LMM (0.6%)
		In_0.83_Al_0.17_As	LMM (1.1%)
		InAs_0.65_P_0.35_	LMM (1.1%)
	*E*_*C*_ = 0.93 eV	InAs_0.35_P_0.45_Sb_0.2_	LM
		Al_0.14_Ga_0.86_Sb	LMM (0.7%)
		InSb_0.15_P_0.85_	LMM (1.6%)
		In_0.73_Al_0.27_As	LMM (1.7%)
		InAs_0.42_P_0.58_	LMM (1.8%)
	*E*_*B*_ = 1.12 eV	InAs_0.13_P_0.6_Sb_0.27_	LM
		Al_0.36_Ga_0.64_Sb	LMM (0.9%)
		In_0.65_Al_0.35_As	LMM (2.3%)
		InSb_0.08_P_0.92_	LMM (2.3%)
		InAs_0.23_P_0.77_	LMM (2.4%)
	*E*_*E*_ = 1.34 eV	Al_0.53_Ga_0.47_Sb	LMM (1.0%)
		In_0.56_Al_0.44_As	LMM (2.8%)
InP	*E*_*A*_ = 0.52 eV	In_0.72_Ga_0.28_As	LMM (1.3%)
		In_0.92_Al_0.18_As	LMM (2.7%)
		InAs_0.83_P_0.17_	LMM (2.7%)
	*E*_*D*_ = 0.70 eV	In_0.44_Ga_0.56_As_0.91_Sb_0.09_	LM
		In_0.05_Ga_0.95_As_0.55_Sb_0.45_	LM
		In_0.57_Ga_0.43_As	LMM (0.2%)
		In_0.83_Al_0.17_As	LMM (2.1%)
		InAs_0.65_P_0.35_	LMM (2.1%)
		InSb_0.25_P_0.75_	LMM (2.6%)
	*E*_*C*_ = 0.93 eV	In_0.7_Ga_0.3_As_0.64_P_0.36_	LM
		In_0.53_Ga_0.31_Al_0.16_As	LM
		In_0.36_Ga_0.64_As	LMM (1.2%)
		InAs_0.42_P_0.58_	LMM (1.3%)
		In_0.73_Al_0.27_As	LMM (1.4%)
		GaAs_0.71_Sb_0.29_	LMM (1.5%)
		InSb_0.15_P_0.85_	LMM (2.2%)
	*E*_*B*_ = 1.12 eV	In_0.84_Ga_0.16_As_0.34_P_0.66_	LM
		In_0.53_Ga_0.18_Al_0.29_As	LM
		InAs_0.23_P_0.77_	LMM (0.7%)
		InSb_0.25_P_0.75_	LMM (0.8%)
		In_0.65_Al_0.35_As	LMM (0.9%)
		In_0.21_Ga_0.79_As	LMM (2.2%)
		GaAs_0.84_Sb_0.16_	LMM (2.5%)
	*E*_*E*_ = 1.34 eV	In_0.99_Ga_0.01_As_0.02_P_0.98_	LM
		In_0.52_Ga_0.06_Al_0.42_As	LM
		InAs_0.01_P_0.99_	LMM ( < 0.1%)
		InSb_0.01_P_0.99_	LMM (0.1%)
		In_0.56_Al_0.44_As	LMM (0.3%)
II-VI on III-V or IV substrate			
GaAs	*E*_*B*_ = 1.12 eV	CuIn(Te_0.34_S_0.66_)_2_	LMM (1.5%)
		CuIn(Se_0.83_S_0.17_)_2_	LMM (1.5%)
		CuIn_0.83_Ga_0.17_Se_2_	LMM (1.8%)
	*E*_*E*_ = 1.34 eV	CuIn(Se_0.37_S_0.63_)_2_	LMM (0.6%)
		CuIn_0.45_Ga_0.55_Se_2_	LMM (0.7%)
		CuIn(Te_0.13_S_0.87_)_2_	LMM (0.8%)
		CuIn_0.75_Al_0.25_Se_2_	LMM (1.5%)
Ge	*E*_*B*_ = 1.12 eV	CuIn(Te_0.34_S_0.66_)_2_	LMM (1.4%)
		CuIn(Se_0.83_S_0.17_)_2_	LMM (1.4%)
		CuIn_0.83_Ga_0.17_Se_2_	LMM (1.7%)
		CuIn_0.93_Al_0.07_Se_2_	LMM (2.0%)
	*E*_*E*_ = 1.34 eV	CuIn_0.45_Ga_0.55_Se_2_	LMM (0.6%)
		CuIn(Se_0.37_S_0.63_)_2_	LMM (0.7%)
		CuIn(Te_0.13_S_0.87_)_2_	LMM (0.9%)
		CuIn_0.75_Al_0.25_Se_2_	LMM (1.4%)
Other configurations			
Si	*E*_*B*_ = 1.12 eV	Si	
Any	*E*_*B*_ = 1.12 eV	CuIn_0.93_Al_0.07_Se_2_	
		CuIn_0.83_Ga_0.17_Se_2_	
		CuIn(Se_0.83_S_0.17_)_2_	
		CuIn(Te_0.34_S_0.66_)_2_	
	*E*_*E*_ = 1.34 eV	CuIn_0.75_Al_0.25_Se_2_	
		CuIn_0.45_Ga_0.55_Se_2_	
		CuIn(Se_0.37_S_0.63_)_2_	
		CuIn(Te_0.13_S_0.87_)_2_	

Proposition of materials to compose the bottom junction of MJSC according to the optimum values for *E*_*G*_^*J1*^. The percentage of lattice mismatching according to the reference substrate is indicated in brackets.

## Conclusions

In conclusion, we have shown that the bandgap energy combinations leading to high efficiencies in optically and electrically series-connected MJSC are abundant. For the AM0 spectrum, the efficiency shows a single peak on the bandgap energy domain whereas for terrestrial spectra a multi-peak distribution is observed. In general, the peaks are redshifted in respect to the bandgap energies and increase with the number of junctions. This is expected due to a well-balanced division of solar photons among the junctions, reducing losses by transmission and thermalization. For a given number of junctions, the efficiency increases logarithmically with concentration, as expected, and the efficiency distribution is also redshifted. We have shown that this is due to the fact that the device configurations presenting lower bandgap energies have steeper increases in the efficiency with the increase of the concentration factor, which bring benefits for its use at high concentrations.

Additionally, we have shown that the optimum values for the bandgap energy of the bottom junction are recurrent for terrestrial spectra at energies close to *E*_*A*_ = 0.52 eV, *E*_*B*_ = 0.70 eV, *E*_*C*_ = 0.93 eV, *E*_*D*_ = 1.12 eV, and *E*_*E*_ = 1.34 eV, matching the high energy edges of the atmospheric absorption bands. Moreover, when the concentration factor changes, the MJSC peak efficiency shifts but the optimum value for the bottom junction bandgap energy is always within the set of recurrent values described before.

Based on the overall results, we have suggested more than a hundred promising III-V, II-VI, and IV semiconductor materials to compose this junction and to serve as basis for the stacking of junctions in a MJSC for any terrestrial application. Silicon is a feature from this list. The increasing interest in integrating III-V, II-VI and perovskites to silicon seems to be a very promising way for further photovoltaic conversion efficiency improvements and cost reduction. We highlight the use of modern techniques, such as direct bonding and (inverted) metamorphic growth, to allow the mechanical integration of what would be formerly called incompatible materials. On the other hand, innovations in the material growth, such as the reuse of substrates, high quality heteroepitaxy, the use of higher growth rates and the use of more efficient processes in the incorporation of precursors, support further reduction in the cost production. The use of such techniques associated with a better choice of materials, based on the results of this work, can push multijunction solar cells to the next level, surpassing the 50% efficiency barrier in a very near future, thus, decreasing costs and making III-V photovoltaics more affordable.

## Methods

### Detailed balance model applied to MJSC

The conversion efficiency of solar cells was herein calculated using the detailed balance model (DBM)^16^ through numerical methods. In this model, the electrical current *I* flowing through a photovoltaic device results from the balance between generation *G* and recombination *R*, as in Eq. (2).

2 $$I=q(G-R)$$

in which *q* is the electronic charge. To calculate the ultimate efficiency of solar cells by the DBM, some assumptions are commonly made and were used in this work: (i) each absorbed photon generates only one electron-hole pair; (ii) each electron-hole pair that recombines only generates one photon; (iii) there is only radiative recombination taking place; (iv) electrical carrier mobility is infinite; (v) material absorptivity A(*E*) is unity for photons with energy *E* higher than *E*_*G*_ and zero otherwise; (vi) there is no reflection at front surface; (vii) the back-surface is perfectly reflective; and (viii) emitted photons can only leave through front surface in which no angle restrictor is applied. Assumptions (iv) and (v) lead to a quantum efficiency *QE*(*E*) of one for photons with energy *E* higher than *E*_*G*_ and zero otherwise. Assumptions (vii) and (viii) mean that the device luminescence is spread out solely to the full upper hemisphere, and lateral emission is neglected.

The application of the DBM to an optically and electrically series-connected MJSC with *N* junctions is straightforward. First, we have to consider that each junction J_i_ (*i* = 1..N increasing from the bottom junction upwards) of bandgap energy ${E}_{G}^{{J}_{i}}$ is subjected to a different incident photon flux ${\rho }_{\gamma }^{i}(\lambda )$ which is filtered from the solar spectrum by the set of junctions above. With the bandgap energies increasing from J_1_ upwards and considering only normal incidence, i.e. *θ* = 0, ${\rho }_{\gamma }^{i}(\lambda )$ can be described as in Eq. (3a):

3a $${\rho }_{\gamma }^{i}(\lambda )=\{\begin{array}{ll}0, & \lambda < {\lambda }_{Ga{p}_{i+1}}\\ {\rho }_{\gamma }^{i+1}(\lambda ), & \lambda \ge {\lambda }_{Ga{p}_{i+1}}\end{array},\,(i=1..N)$$

3b $${\rho }_{\gamma }^{N+1}(\lambda )=\frac{{W}_{sun}(\lambda )}{hc/\lambda }$$

In Eq. (3a), ${\lambda }_{Ga{p}_{i}}$ is the material cutoff wavelength (corresponding to ${E}_{G}^{{J}_{i}}$) of J_i_, and ${\lambda }_{Ga{p}_{i+1}}$ is the material cutoff wavelength of J_i+1_, the junction immediately above J_i_. In the case of the uppermost junction J_N_, ${\lambda }_{Ga{p}_{N+1}}=0$ shall be considered. Equation (3b) defines ${\rho }_{\gamma }^{N+1}(\lambda )$ as the solar photon flux calculated from the solar spectral irradiance ${W}_{sun}(\lambda )$, using *h* for the Planck constant, and *c* and *λ* as the speed and wavelength of light, respectively.

In this context, each junction J_i_ has its own generation rate *G*_*i*_ and recombination rate *R*_*i*_. *G*_*i*_ is the temporal rate of absorbed photons per unit of area in J_i_, which depends upon the incident photon flux and the quantum efficiency (*QE*_*i*_), as in Eq. (4):

4 $${G}_{i}={\int }_{0}^{\infty }Q{E}_{i}(\lambda ){\rho }_{\gamma }^{i}(\lambda )d\lambda =\,{\int }_{{\lambda }_{Ga{p}_{i+1}}}^{{\lambda }_{Ga{p}_{i}}}[\frac{{W}_{sun}(\lambda )}{hc/\lambda }]d\lambda $$

*R*_*i*_ can be calculated by the temporal rate of photons spontaneously emitted by J_i_ per unit of area through radiative recombination. Let ${\rho }_{BB}^{i}(\lambda ,V,T)$ denotes the blackbody spectral photon flux at temperature *T* under applied voltage *V*, and ${A}_{i}(\lambda )$ the material absorptivity of J_i_. Then, *R*_*i*_ can be expressed as in Eq. (5)^17^:

5 $${R}_{i}(V)={\int }_{0}^{{\theta }_{E}}\,{\int }_{0}^{\infty }{A}_{i}(\lambda ){\rho }_{BB}^{i}(\lambda ,V,T)d\lambda d\varOmega =2\pi {\int }_{0}^{{\lambda }_{Ga{p}_{i}}}\frac{2c}{{\lambda }^{4}}\frac{d\lambda }{[{e}^{(\frac{hc}{\lambda }-q\cdot V)/kT}-1]}$$

in which ${\theta }_{E}=2\pi$ sterradian is the solid angle of emission (full upper hemisphere). The first equalities in Eqs. (4) and (5) are valid in a general case whereas the second ones are obtained using assumptions (i) to (viii) and Eq. (3).

The application of the DBM to a MJSC was carried out by considering the electrical series-connection constraint. Thus, the same electrical current *I*_*MJSC*_, which is the least of the *N* junctions, was imposed to flow through the junctions (Eq. 6) whereas the total voltage of the *N* series-connected generators at each point was summed (Eq. 7).

6 $${I}_{MJSC}(V)={I}_{min}(V)={{\rm{\min }}}_{i=1..N}[{I}_{i}(V)]$$

7 $${V}_{MJSC}({I}_{MJSC})={\sum }_{i=1}^{N}{V}_{i}({I}_{MJSC})$$

The relevant part of the IV characteristic was accessed by sweeping the parameter *V* in the range:

8 $$-{\sum }_{i=1}^{N}{V}_{O{C}_{i}}\le V\le {V}_{O{C}_{low}}$$

In Eq. (8), $\,{V}_{O{C}_{i}}$ and ${V}_{O{C}_{low}}$ are the open circuit voltage of J_i_ and of the junction with the lowest current density, respectively.

Finally, from the described procedure, the figures of merit, such as the short-circuit current density ${I}_{SC}^{MJSC}$, the open circuit voltage ${V}_{OC}^{MJSC}$, and the fill factor $F{F}_{MJSC}$, are extracted from the calculated MJSC IV curve. Using the incident power *P*_*in*_, the efficiency ${\eta }_{MJSC}$ can be calculated with Eq. (9).

9 $${\eta }_{MJSC}=\frac{{P}_{MPP}^{MJSC}}{{P}_{in}}=\frac{F{F}_{MJSC}\cdot {I}_{SC}^{MJSC}\cdot {V}_{OC}^{MJSC}}{{P}_{in}}$$

In our study, standard ASTM solar spectra^61^ are used and temperature is set to T = 25 °C. No optical coupling was assumed, which means that the luminescence coming from the radiative recombination of a junction is not used by the other ones. If so, Eqs. (4) and (5) would be coupled between the *N* junctions, as *G*_*i*_ would depend on the several *R*_*i’*_ (i′ > i). Under this condition, the method of calculating the total MJSC IV curve by the application of Eqs. (6) and (7) for *N* IV curves obtained independently could not be followed. Nonetheless, as Martí and Araujo^20^ discuss, the limiting efficiency is higher for a situation with no luminescence coupling in which perfect filters are put in between the junctions in order to reflect the luminescence only allowing it to exit from the front side. This reflects the case under consideration here.

The model has been validated through the comparisons of the results with other theoretical studies. Details can be found in the Supplementary Material.

### Search methodology for the highest efficiency of MJSC

The procedure described above correlates a particular set of bandgap energies of a MJSC to its figures of merit. The search for the highest efficiencies for each device condition is performed by sweeping up the possible bandgap energy combinations in the *N*-dimensional space. However, the best configuration is expected to be reached for a singular condition: when the maximum power point current of the junctions ${I}_{MP{P}_{i}}$ is the same^55^. As a first estimative for the best candidate to the highest efficiency, we defined a simple test to be performed prior to the entire calculation. Instead of using the equality of the ${I}_{MP{P}_{i}}$, which can only be performed after the calculation of the *N* IV curves, we used the generation current in each junction ${I}_{{G}_{i}}$ as the parameter to be equalized, which can be obtained directly from a simple analysis of the solar spectrum. This is justified by the great similarity between such values, as recombination at the maximum power point is normally very low. Thus, for a given bandgap energy of the bottom junction *E*_*G*_^*J1*^ (which defines its cutoff wavelength ${\lambda }_{Ga{p}_{1}}$), an optimized maximum power point current density ${I}_{MPP}^{MJSC}$ would be obtained by a perfectly balanced division of solar photons through the *N* junctions, as Eq. (10) describes:

10 $${I}_{MPP}^{MJSC}\approx \frac{{\sum }_{i=i}^{N}{I}_{{G}_{i}}}{N}=\frac{q}{N}{\int }_{0}^{{\lambda }_{Ga{p}_{1}}}[\frac{{W}_{sun}(\lambda )}{hc/\lambda }]\,d\lambda $$

After this, the best candidate is found by calculating the bandgap energies leading to the same currents ${I}_{{G}_{i}}={I}_{MPP}^{MJSC}$ through Eq. (11):

11 $$q{\int }_{0}^{{\lambda }_{Ga{p}_{N}}}[\frac{{W}_{sun}(\lambda )}{hc/\lambda }]\,d\lambda =q{\int }_{{\lambda }_{Ga{p}_{N}}}^{{\lambda }_{Ga{p}_{N-1}}}[\frac{{W}_{sun}(\lambda )}{hc/\lambda }]\,d\lambda =\ldots =q{\int }_{{\lambda }_{Ga{p}_{2}}}^{{\lambda }_{Ga{p}_{1}}}[\frac{{W}_{sun}(\lambda )}{hc/\lambda }]\,d\lambda ={I}_{MPP}^{MJSC}$$

The first estimative is then the starting point for the search of the best bandgap energy combination leading to the highest efficiencies. Under all conditions tested in this work concerning number of junctions (from 2 to 6) and illumination (AM0, AM1.5 g, AM1.5d under concentrations up to 3000 suns), the maximum difference between the correct bandgap energies leading to the highest efficiencies and the ones from the estimative is less than 0.05 eV and the difference between the optimum and the estimated value for ${I}_{MPP}^{MJSC}$ is less than 5%. The sweeping resolution in the bandgap energy was 0.01 eV in all optimization processes performed in this work.

## Supplementary information

Supplementary Information

## Data Availability

The datasets generated by the current study are available from the corresponding author on reasonable request.
