# Supplementary material for: The Influence of Solar Spectrum and Concentration Factor on the Material Choice and the Efficiency of Multijunction Solar Cells
Source: Sci Rep. 2019 Dec 27;9:20055. doi: 10.1038/s41598-019-56457-0 (PMC6934521; doi:10.1038/s41598-019-56457-0)
Supplement: Supplementary file 1 — Supplementary Information [file 41598_2019_56457_MOESM1_ESM.pdf]

The Influence of Solar Spectrum and Concentration Factor on the Material Choice and Efficiency of Multijunction Solar Cells

Supplementary Material

*Daniel N. Micha[1], Ricardo T. Silvares Junior[1]

[1]Centro Federal de Educação Tecnológica Celso Suckow da Fonseca, CEFET/RJ, Rio de Janeiro 20271-110, Brazil.

*daniel.micha@cefet-rj.br

Validation of the model and the results

The model used in our simulations has been validated through the comparison of the results shown in this work (R_{work}) with the ones from two references (R_{ref}): Nozik et al. [1] and Zhu et al. [2]. Table S1 shows the efficiencies and bandgap energies of the best MJSC for several configurations. Additionally, the discrepancy in our results when compared to the references, as defined in equation (S1), is shown.

$$\frac{\delta R}{R} = \left|1 - \frac{R_{work}}{R_{ref}}\right| \qquad \text{(S1)}$$

Table S1 | Validation of the theoretical model. Comparison of the highest efficiency and optimum bandgap energy combination for several applications as obtained with the model used in this work and the ones from Nozik et al. [1] and Zhu et al. [2].

Configuration	This work	Ref. [1]	$\delta R(1)$ [%]	Ref. [2]	$\delta R(2)$ [%]
2JSC @ AM0	41.6%	42.2%	1.4	-	-
	E_G^{J1} = 0.93 eV	E_G^{J1} = 0.89 eV	4.5	-	-
	E_G^{J2} = 1.62 eV	E_G^{J2} = 1.58 eV	2.5	-	-
2JSC @ AM1.5g	44.9%	45.9%	2.2	44.6%	0.7
	E_G^{J1} = 0.96 eV	E_G^{J1} = 0.93 eV	3.2	E_G^{J1} = 0.94 eV	2.1
	E_G^{J2} = 1.63 eV	E_G^{J2} = 1.60 eV	1.2	E_G^{J2} = 1.58 eV	3.2
2JSC @ 500 x AM1.5d	52.6%	53.8%	2.2	-	-
	E_G^{J1} = 0.93 eV	E_G^{J1} = 0.93 eV	0.0	-	-
	E_G^{J2} = 1.57 eV	E_G^{J2} = 1.57 eV	0.0	-	-
2JSC @ 1000 x AM1.5d	53.5%	-	-	53.2%	0.6
	E_G^{J1} = 0.93 eV	-	-	E_G^{J1} = 0.93 eV	0.0
	E_G^{J2} = 1.57 eV	-	-	E_G^{J2} = 1.55 eV	1.3
3JSC @ AM0	47.8%	48.5%	1.4		-
	E_G^{J1} = 0.77 eV	E_G^{J1} = 0.74 eV	4.1	-	-
	E_G^{J2} = 1.21 eV	E_G^{J2} = 1.18 eV	2.5	-	-
	E_G^{J3} = 1.84 eV	E_G^{J3} = 1.82 eV	1.1	-	-
3JSC @ AM1.5g	50.7%	51.8%	2.1	50.2%	1.0
	E_G^{J1} = 0.93 eV	E_G^{J1} = 0.93 eV	0.0	E_G^{J1} = 0.93 eV	0.0
	E_G^{J2} = 1.36 eV	E_G^{J2} = 1.37 eV	0.7	E_G^{J2} = 1.35 eV	0.7
	E_G^{J3} = 1.90 eV	E_G^{J3} = 1.90 eV	0.0	E_G^{J3} = 1.88 eV	1.1

3JSC @ 500 x AM1.5d	59.5%	61%	2.5	-	-
	E_G^{J1} = 0.70 eV	E_G^{J1} = 0.69 eV	1.4	-	-
	E_G^{J2} = 1.18 eV	E_G^{J2} = 1.17 eV	0.9	-	-
	E_G^{J3} = 1.75 eV	E_G^{J3} = 1.74 eV	0.6	-	-
3JSC @ 1000 x AM1.5d	60.6%	-	-	60.0%	1.0
	E_G^{J1} = 0.70 eV	-	-	E_G^{J1} = 0.70 eV	0.0
	E_G^{J2} = 1.18 eV	-	-	E_G^{J2} = 1.16 eV	1.7
	E_G^{J3} = 1.75 eV	-	-	E_G^{J3} = 1.73 eV	1.2
4JSC @ AM0	51.5%	52.3%	1.5	-	-
	E_G^{J1} = 0.68 eV	E_G^{J1} = 0.65 eV	4.6	-	-
	E_G^{J2} = 1.01 eV	E_G^{J2} = 0.99 eV	2.0	-	-
	E_G^{J3} = 1.42 eV	E_G^{J3} = 1.40 eV	1.4	-	-
	E_G^{J4} = 2.01 eV	E_G^{J4} = 1.99 eV	1.0	-	-
4JSC @ AM1.5g	54.3%	55.6%	2.3	53.5%	
	E_G^{J1} = 0.72 eV	E_G^{J1} = 0.72 eV	0.0	E_G^{J1} = 0.72 eV	
	E_G^{J2} = 1.12 eV	E_G^{J2} = 1.11 eV	0.9	E_G^{J2} = 1.11 eV	
	E_G^{J3} = 1.50 eV	E_G^{J3} = 1.49 eV	0.7	E_G^{J3} = 1.48 eV	
	E_G^{J4} = 2.01 eV	E_G^{J4} = 2.00 eV	0.5	E_G^{J4} = 1.98 eV	
4JSC @ 500 x AM1.5d	63.4%	65.1%	2.6	-	-
	E_G^{J1} = 0.53 eV	E_G^{J1} = 0.51 eV	3.9	-	-
	E_G^{J2} = 0.97 eV	E_G^{J2} = 0.96 eV	1.0	-	-
	E_G^{J3} = 1.38 eV	E_G^{J3} = 1.37 eV	0.7	-	-
	E_G^{J4} = 1.89 eV	E_G^{J4} = 1.88 eV	0.5	-	-
4JSC @ 1000 x AM1.5d	64.5%	-	-	63.8%	2.7
	E_G^{J1} = 0.51 eV	-	-	E_G^{J1} = 0.52 eV	2.0
	E_G^{J2} = 0.96 eV	-	-	E_G^{J2} = 0.95 eV	1.1
	E_G^{J3} = 1.37 eV	-	-	E_G^{J3} = 1.35 eV	1.5
	E_G^{J4} = 1.88 eV	-	-	E_G^{J4} = 1.85 eV	1.6
5JSC @ AM0	54.0%	54.8%	1.5	-	-
	E_G^{J1} = 0.64 eV	E_G^{J1} = 0.64 eV	0.0	-	-
	E_G^{J2} = 0.91 eV	E_G^{J2} = 0.91 eV	0.0	-	-
	E_G^{J3} = 1.22 eV	E_G^{J3} = 1.21 eV	0.8	-	-
	E_G^{J4} = 1.60 eV	E_G^{J4} = 1.60 eV	0.0	-	-
	E_G^{J5} = 2.15 eV	E_G^{J5} = 2.15 eV	0.0	-	-
5JSC @ AM1.5g	56.5%	57.9%	2.4	55.8%	1.3
	E_G^{J1} = 0.70 eV	E_G^{J1} = 0.70 eV	0.0	E_G^{J1} = 0.70 eV	0.0
	E_G^{J2} = 1.00 eV	E_G^{J2} = 1.01 eV	1.0	E_G^{J2} = 0.99 eV	1.0
	E_G^{J3} = 1.31 eV	E_G^{J3} = 1.33 eV	1.5	E_G^{J3} = 1.27 eV	3.1
	E_G^{J4} = 1.66 eV	E_G^{J4} = 1.67 eV	0.6	E_G^{J4} = 1.64 eV	1.2
	E_G^{J5} = 2.14 eV	E_G^{J5} = 2.14 eV	0.0	E_G^{J5} = 2.11 eV	1.4
5JSC @ 500 x AM1.5d	65.6%	67.5%	2.8	-	-
	E_G^{J1} = 0.57 eV	E_G^{J1} = 0.54 eV	5.6	-	-
	E_G^{J2} = 0.95 eV	E_G^{J2} = 0.92 eV	3.3	-	-
	E_G^{J3} = 1.23 eV	E_G^{J3} = 1.21 eV	1.6	-	-
	E_G^{J4} = 1.58 eV	E_G^{J4} = 1.56 eV	1.3	-	-
	E_G^{J5} = 2.05 eV	E_G^{J5} = 2.03 eV	1.0	-	-
5JSC @ 1000 x AM1.5d	66.7%	-	-	66.2%	0.8
	E_G^{J1} = 0.57 eV	-	-	E_G^{J1} = 0.55 eV	3.6
	E_G^{J2} = 0.94 eV	-	-	E_G^{J2} = 0.92 eV	2.2
	E_G^{J3} = 1.22 eV	-	-	E_G^{J3} = 1.20 eV	1.7

	E_G^{J4} = 1.57 eV	-	-	E_G^{J4} = 1.55 eV	1.3
	E_G^{J5} = 2.04 eV	-	-	E_G^{J5} = 2.02 eV	1.0
6JSC @ AM0	55.8%	56.7%	1.6	-	-
	E_G^{J1} = 0.58 eV	E_G^{J1} = 0.59 eV	1.7	-	-
	E_G^{J2} = 0.82 eV	E_G^{J2} = 0.83 eV	1.2	-	-
	E_G^{J3} = 1.06 eV	E_G^{J3} = 1.07 eV	0.9	-	-
	E_G^{J4} = 1.35 eV	E_G^{J4} = 1.36 eV	0.7	-	-
	E_G^{J5} = 1.72 eV	E_G^{J5} = 1.73 eV	0.6	-	-
	E_G^{J6} = 2.25 eV	E_G^{J6} = 2.26 eV	0.4	-	-
6JSC @ AM1.5g*	57.7%	59.2%	2.5	-	-
	E_G^{J1} = 0.52 eV	E_G^{J1} = 0.51 eV	2.0	-	-
	E_G^{J2} = 0.80 eV	E_G^{J2} = 0.79 eV	1.3	-	-
	E_G^{J3} = 1.13 eV	E_G^{J3} = 1.13 eV	0.0	-	-
	E_G^{J4} = 1.41 eV	E_G^{J4} = 1.41 eV	0.0	-	-
	E_G^{J5} = 1.74 eV	E_G^{J5} = 1.73 eV	0.6	-	-
	E_G^{J6} = 2.20 eV	E_G^{J6} = 2.19 eV	0.5	-	-
6JSC @ 500 x AM1.5d	67.4%	69.1%	2.5	-	-
	E_G^{J1} = 0.52 eV	E_G^{J1} = 0.53 eV	1.9	-	-
	E_G^{J2} = 0.80 eV	E_G^{J2} = 0.79 eV	1.3	-	-
	E_G^{J3} = 1.13 eV	E_G^{J3} = 1.11 eV	1.8	-	-
	E_G^{J4} = 1.40 eV	E_G^{J4} = 1.38 eV	1.4	-	-
	E_G^{J5} = 1.71 eV	E_G^{J5} = 1.69 eV	1.2	-	-
	E_G^{J6} = 2.15 eV	E_G^{J6} = 2.13 eV	0.9	-	-

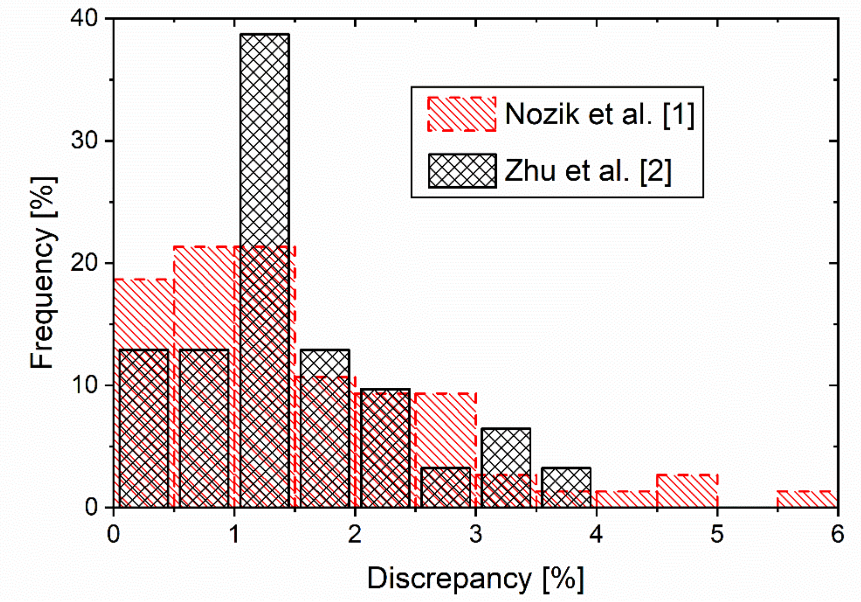

Figure S1 | Validation of the theoretical model. Frequency of the discrepancy between the results of this work and the ones from Nozik et al. [1] and from Zhu et al. [2].

Fig. S1 presents a histogram of the discrepancy, as defined in equation (S1), between the results obtained from this work and the ones from Nozik et al. [1] and the ones from Zhu et al. [2] It is possible to see that the discrepancy has peaks between 1.0% and 1.5% and that 90% of

the data have discrepancies lower than 2.5%, which is a good result. Only one result (0.9%) was distant from the reference with a discrepancy higher than 5%.

References

[1] Nozik A.J., Conibeer G. & Beard M.C.(Eds.). *Advanced concepts in photovoltaics* (Royal Society of Chemistry, 2014).

[2] Zhu L. et al. Conversion efficiency limits and bandgap designs for multi-junction solar cells with internal radiative efficiencies below unity. *Optics Express* **24**, A740-A751 (2016).
